# Unconventional Myosins from *Caenorhabditis elegans* as a Probe to Study Human Orthologues

**DOI:** 10.3390/biom12121889

**Published:** 2022-12-16

**Authors:** Chloe A Johnson, Ranya Behbehani, Folma Buss

**Affiliations:** Cambridge Institute for Medical Research, University of Cambridge, Hills Road, Cambridge CB2 0XY, UK

**Keywords:** myosin, motor protein, nematode, model organism

## Abstract

Unconventional myosins are a superfamily of actin-based motor proteins that perform a number of roles in fundamental cellular processes, including (but not limited to) intracellular trafficking, cell motility, endocytosis, exocytosis and cytokinesis. 40 myosins genes have been identified in humans, which belong to different 12 classes based on their domain structure and organisation. These genes are widely expressed in different tissues, and mutations leading to loss of function are associated with a wide variety of pathologies while over-expression often results in cancer. *Caenorhabditis elegans* (*C. elegans*) is a small, free-living, non-parasitic nematode. ~38% of the genome of *C. elegans* has predicted orthologues in the human genome, making it a valuable tool to study the function of human counterparts and human diseases. To date, 8 unconventional myosin genes have been identified in the nematode, from 6 different classes with high homology to human paralogues. The *hum-1* and *hum-5* (heavy chain of an unconventional myosin) genes encode myosin of class I, *hum-2* of class V, *hum-3* and *hum-8* of class VI, *hum-6* of class VII and *hum-7* of class IX. The *hum-4* gene encodes a high molecular mass myosin (307 kDa) that is one of the most highly divergent myosins and is a member of class XII. Mutations in many of the human orthologues are lethal, indicating their essential properties. However, a functional characterisation for many of these genes in *C. elegans* has not yet been performed. This article reviews the current knowledge of unconventional myosin genes in *C. elegans* and explores the potential use of the nematode to study the function and regulation of myosin motors to provide valuable insights into their role in diseases.

## 1. Introduction

Myosins are ubiquitously expressed, multifunctional motor proteins that converts the energy generated from the hydrolysis of ATP to facilitate movement. They are involved in a wide range of cellular processes including cargo transport, formation of actin-based projections at the plasma membrane, steady state distribution of membrane compartments, cytokinesis, and muscle contraction. Myosins share a common domain organisation comprised of head, neck and tail regions [1]. The myosin head is composed of the motor domain, which contains the sites for catalytic activity, and the neck region, which functions as the lever arm. This neck region contains a variable number of IQ motifs, which are units of 23 amino acids with the sequence IQXXXRGXXXR that serve as a binding site for calmodulin, or calmodulin-like proteins such as myosin light chains [2,3]. The myosin superfamily is traditionally grouped into conventional class II myosins and the more divergent unconventional myosins. Conventional myosins are characterised by a tail region consisting of a coiled-coil forming sequence which promotes homodimer formation of long-rod structured tails that self-associate to form bipolar filaments [4,5,6]. The remaining myosins are the unconventional myosins with highly diverse tails, which bind either directly or through adaptor proteins to various cargoes including but not limited to membrane vesicles, organelles, larger protein complexes or ribonucleoproteins [7,8,9]. Thus, myosin motors are essential in many biological processes such as intracellular trafficking, mechanical support and force sensing.

The functional diversity of myosin motors is reflected in the large number of genes within this superfamily. At least 35 classes of myosins have been identified in eukaryotes, 12 of which are found in the human genome [10]. Class I and class II myosins are proposed to be the most ancient [11]; indeed, all eukaryotic animal cells examined contain at least one myosin II gene and multiple myosin I genes. In addition, myosins of class V are found widely, if not universally. The simple model organisms *Saccharomyces cerevisiae* and *Schizosaccharomyces pombe* both encode genes for myosins from class I, II and V [12,13,14,15,16]. The multicellular, complex model organisms *Drosophila melanogaster* and *Caenorhabditis elegans* express in addition to the basic myosin repertoire of I, II and V also myosins from class VI and VII [17,18,19]. All of these myosin classes have been implicated in diseases in humans, such as cancer, hearing loss, neurodegeneration and myopathies [20]. Many of the disease mechanisms remain to be established and therefore, a model organism with orthologues of these human disease-causing genes is vital for elucidating disease pathogenesis.

**Figure 1:**
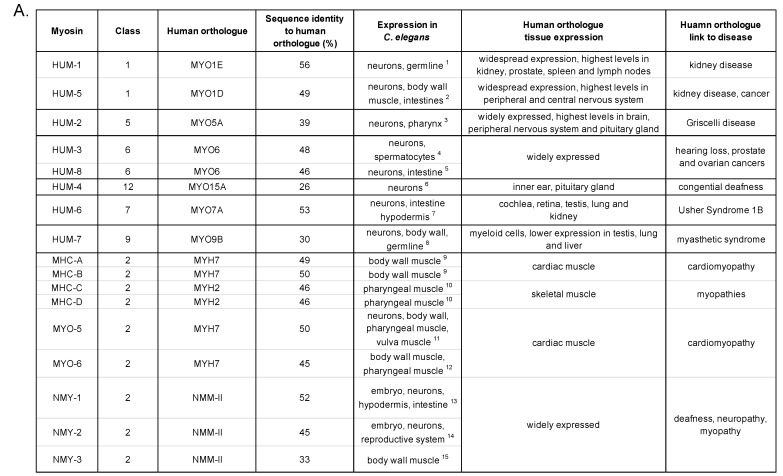

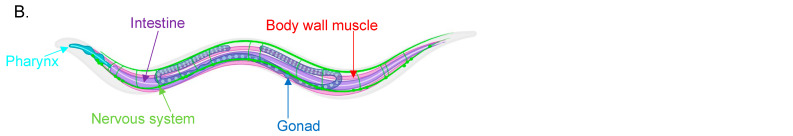
(**A**) Table providing an overview of myosin genes and their properties found in *C. elegans*. ^1^ [24,25] ^2^ [25,26,27] ^3^ [28,29,30] ^4^ [30,31,32] ^5^ [24,25] ^6^ [33] ^7^ [25,27,34] ^8^ [25,34,35] ^9^ [36,37] ^10^ [38] ^11^ [26,34,39] ^12^ [34,40,41] ^13^ [26,34,42,43] ^14^ [24,34,42,44] ^15^ [34,40]. (**B**) Schematic of tissue distribution in *C. elegans*. Body wall muscle is shown in red, the nervous system in green, intestines in purple, gonads/reproductive system in blue and pharynx in teal. This figure has been created using https://app.biorender.com (accessed on 25 October 2022).

*C. elegans* is a microscopic soil nematode that is now established as a powerful genetic model organism [21], being studied extensively with respect to development and genetics, cell lineage, and more recently, the role of ageing in human health and disease [22]. This is due to the versatility of the worm with its short life cycle, stereotypical development, small size, and notably, its transparency. In addition, approximately 38% of the *C. elegans* genome has predicted orthologues in the human genome. To date, eight unconventional myosin genes from six different classes with high homology to human paralogues have been identified in the nematode [23] (Figure 1). The *hum-1* and *hum-5* (heavy chain of an unconventional myosin) genes encode members of myosins of class I, *hum-2* of class V, *hum-3* and *hum-8* of class VI, *hum-6* of class VII and *hum-7* of class IX. The *hum-4* gene encodes a high molecular mass myosin (307 kDa) of class XII one of the most highly divergent myosins. Mutations or deletions of most of these myosins in humans are lethal, indicating their essential properties [20]. This review will provide an overview of the current knowledge of myosin genes in *C. elegans* and discuss the potential use of this model organism to study the role of myosin motors in human disease.

## 2. HUM-1 and HUM-5

**Figure 2:**
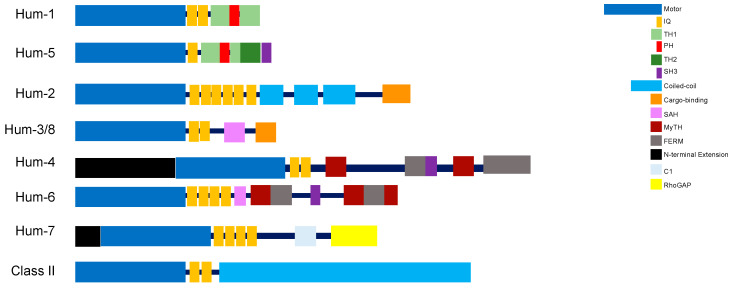
Schematic of the domain organisation of myosin isoforms found in *C. elegans*. All motors are comprised of a head region termed the motor domain, a neck region with variable number of IQ motifs, and highly variable tail regions. Abbreviations are as follows; TH1—tail homology 1; PH—pleckstrin homology; TH2—tail homology 2; SH3—Src homology 3; SAH—single alpha helical; MyTH—myosin tail homology; FERM—4.1 protein-ezrin-radixin-moesin.

Class I myosins are a large and diverse class, comprising various monomeric membrane-associated motor proteins. Higher eukaryotes typically express eight distinct myosin I genes, *MYO1A-MYO1H* [45]. Myosin I motors follow the typical domain organisation with a light-chain binding neck domain that can bind between one to six calmodulins or calmodulin-like light chains, and a tail domain that is comprised of a myosin I family tail homology (TH1) domain, which encompasses a polybasic pleckstrin homology (PH) domain able to bind to anionic phospholipid membranes (Figure 2) [46,47]. Six of the myosin I genes in higher eukaryotes encode short-tailed isoforms (MYO1A-MYO1D, MYO1G, and MYO1H), whilst two encode long-tailed isoforms (MYO1E and MYO1F) that contain additional proline-rich (TH2) and Src homology 3 (SH3/TH3) domains [47]. All myosins of class I have a relatively low duty ratio compared to myosins of other classes [48]. They can be grouped into either fast movers or strain sensors whose activity is regulated by load.

*C. elegans* express two distinct myosins of class I, HUM-1 and HUM-5, encoding proteins of 134 kDa and 117 kDa, respectively [23]. HUM-1 is a homologue of human MYO1E, sharing a 56% sequence identity, while HUM-5 is a homologue of human MYO1D, with 49% sequence identity. HUM-1, a so called long tailed myosin of class I contains a short lever arm with a single IQ motif, whereas HUM-5 has two IQ motifs in the neck domain and a short basic tail [23,49].

HUM-1 is expressed in various tissues, including within the mechanosensory posterior ventral process and outer labial lateral neurons and the *C. elegans* germline [24,25]. Its direct human homologue, MYO1E, is widely expressed and found in most cell types, often at regions of the plasma membrane with dynamic actin structures such as adherens junctions, focal adhesions, and lamellipodia [47,50]. MYO1E is generally involved in the maintenance of plasma membrane tension, the stability of cell–cell adhesion, as well as clathrin-mediated endocytosis [47,51,52]. A HUM-1 knockout worm has been generated (*hum-1*(ok634)) (Caenorhabditis Genetics Center), however, details of associated phenotypes have not yet been described. HUM-5, on the other hand, is expressed in an array of tissues that includes intestinal tissue, body wall muscle, and sensory neurons [25,26,27]. HUM-5 has also been identified as a potential regulator of axon guidance [53]. Its mammalian homologue, MYO1D, is also highly expressed in the central and peripheral nervous systems and has been suggested to play a role in the fusion of early endosomes and the maintenance of plasma membrane tension ([54,55,56]. As with HUM-1, a HUM-5 knockout has been generated (*hum-5*(ok1885)), but no phenotypic analyses have yet been published on the strain.

The human orthologues of HUM-1 and HUM-5, MYO1E and MYO1D, respectively, have been implicated in a variety of diseases. A missense mutation in MYO1E causes focal segmental glomerulosclerosis and a homozygous truncation mutation in the motor domain of MYO1E has been linked to nephrotic syndrome, both childhood—onset forms of kidney disease [57,58]. Increased MYO1E expression has also been found to play a role in metastasis of breast cancers [59], whereas MYO1D expression has been shown to be mis-regulated in several prostate cancers [60].

## 3. HUM-2

The *hum-2* gene is an orthologue of human MYO5A, a class V myosin [23]. Humans contain three genes for myosins of class V, which express MYO5A, MYO5B and MYOV5C, all of which are alternatively spliced to produce further isoforms. Genes for class V myosins are also present in other model organisms; *Drosophila* contain a single MYO5 gene called *didum*, while the yeasts *Saccharomyces cerevesiae* and *Schizosaccharomyces pombe* both have two MYO5 genes, *MYO2* and *MYO4*, and *myo51* and *myo52,* respectively [12,61]. HUM-2 shares a 39% sequence identity with human MYO5A, 38% with *Drosophila didum,* and 30–33% with the yeast myosins of class V. HUM-2 contains a very long neck region with six IQ motifs, which is common to all class V myosins, and allows a large step size of 36 nm, the pitch of the helical actin filament. The tail region contains three coiled-coil domains, important for dimerization, and a globular tail domain, which has been shown to enable cargo binding in other members of class V myosins (Figure 2). There is divergence in the sequence of HUM-2 and human MYO5 throughout the length of the motor protein.

Myosins of class V form a dimer and display processive movement in a hand-over-hand fashion. They are kinetically adapted to move processively, because of their large working stroke/step size and high duty ratio, which is the fraction of the time the motor is attached to an actin filament during one complete cycle [62,63]. MYO5A is widely expressed in many tissues, including the brain, peripheral nervous system and pituitary gland [64]. Whilst the kinetic and motile properties of HUM-2 have yet to be elucidated, tissue expression is similar to its human orthologue in a number of different neurons, including dorsal B and D type motor neurons, posterior lateral type N interneuron, and posterior ventral process neurons in both the adult and larval stages [28,29,30]. HUM-2 knock out strains (*hum-2(ok596)*) have been generated (Caenorhabditis Genetics Center), however, no detailed analysis has been performed so far. Mutations in human MYO5A give rise to the rare, autosomal recessive Griscelli Type 1 disease, characterised by neurological impairment [65]. Future studies may utilise HUM-2 and the very well-defined and genetically tractable nervous system in *C. elegans* to explore basic mechanistic pathways that might be underpin complex human neurological diseases.

## 4. HUM-3 and HUM-8

Myosins of class VI are unique amongst myosin motors in that they move towards the minus end of actin filaments [66]. *C. elegans* possesses two genes for myosins of class VI, *hum-3* (spe-15) and *hum-8*, which are orthologues of the human MYO6 gene. In contrast, humans express a single MYO6 gene that can undergo alternative splicing to produce four different isoforms [67]. Human MYO6 shares a 48% sequence identity with HUM-3, and 45% with HUM-8. The Drosophila genome also encodes a single MYO6, known as *jaguar*, which shares 44% and 39% sequence identities with HUM-3 and HUM-8, respectively. The sequence differences are scattered throughout the protein, but many motifs and regulatory elements are conserved, such as the GESGAGKT sequence of the ATP-binding P-loop, and the phosphorylatable TEDS-rule site (Thr405 in human MYO6) in the motor domain. In the tail, the RRL motif is conserved, whilst the WWY motif is altered in both Hum-3 (MWY) and Hum-8 (MWF). Comparing the sequences of HUM-3 and HUM-8 yields a 63% sequence identity between them, which is enough divergence to suggest functional variation. Yeasts do not possess orthologues to MYO6 in their genome.

HUM-3 and HUM-8 follow a domain organisation typical of class VI myosins, consisting of a motor domain followed by a neck region containing a single IQ motif, and a tail that terminates in a cargo-binding domain (CBD) (Figure 2). Both *C. elegans* MYO6s also have sequences corresponding to the two unique inserts characteristic of myosins of this class: a 53-residue insertion (reverse gear or insert-2) between its converter and light chain-binding domain that is solely responsible for the reverse directionality of MYO6, and a second shorter insert (insert-1) near the nucleotide-binding pocket which has been shown to modulate nucleotide binding [68,69]. Interestingly, unlike human MYO6, both HUM-3 and HUM-8 have an N-terminal extension, 8 and 73 residues, respectively. These extensions have no homology to known domains or motifs, with the precise function yet to be elucidated.

Mammalian MYO6 is monomeric, has a large working stroke (~18 nm) and a high duty ratio, and is weakly processive as a dimer [70,71,72,73]. In the presence of high load or ADP, MYO6 switches from a dimeric transporter to a dynamic tension sensor, translocating along actin to maintain force in a system, and anchoring membrane compartments (e.g., endosomes) to actin filaments [74,75]. The kinetic and biophysical properties of HUM-3 and HUM-8 have yet to be elucidated.

Human MYO6 is widely expressed, including in neuronal tissues, the inner ear hair cells of the cochlea, and the intestine. MYO6 undergoes alternative splicing of two inserts in its CBD: the small insert (SI, adding nine residues) and the large insert (LI, adding 21–31 residues). Human MYO6 has four splice isoforms (holding the SI, LI, both, or none), which have differential interaction networks and binding partners and are expressed in different cell types and tissues [67]. The predominant functions of human MYO6 are linked to endocytosis, protein secretion, autophagy, and the regulation of actin filament dynamics and involve a wide range of adaptor proteins [67,76,77,78,79,80,81]. The expression of HUM-3 and HUM-8 indicates a differential expression pattern between the two worm MYO6s. HUM-3 is present in wide array of neurons, including anterior ventral process class neurons and the neurosecretory motor neuron, as well as in the *C. elegans* germline [30,31,32]. HUM-8, in contrast, is expressed in a few select tissues, such as the intestine and the posterior ventral process neuron [24,25]. Interestingly, the distinct expression of HUM-3 and HUM-8 is seen in tissues analogous to those where the different splice variants of human MYO6 are found.

Very little is known about the functions, biochemical and biophysical properties of either HUM-3 and HUM-8. The disruption of HUM-8 using RNA interference causes cytokinesis defects within the germline, which coincides with the role of human MYO6 in membrane delivery during mammalian cytokinesis [82,83]. HUM-3 on the other hand is essential for spermiogenesis in *C. elegans*, as HUM-3 knockout worms are almost completely self-sterile and display gross cytological defects in the morphology of budding spermatids and the residual body. The spermatids typically fail to activate to form ameboid spermatozoa and are characterised by the improper partitioning of Golgi-derived fibrous body membrane organelles (FB-Mos) and mitochondria [84,85]. Detailed studies have shown that HUM-3 is involved in the final cytokinetic step during spermatid budding, where it assembles into stable ring-like structures that contract to seal cortical actin, constrict the membrane, and promote cytokinesis [86]. Interestingly, mammalian MYO6 is also expressed in mammalian testes, and has been shown to regulate the formation of actin structures and the three-dimensional organisation of membrane compartments during spermatid development [87,88,89].

Mutations in human MYO6 have been linked to several diseases such as hearing loss and familial hypertrophic cardiomyopathy [88,90]. Furthermore, MYO6 overexpression correlates with clinically aggressive behaviour in both ovarian and prostate carcinomas, which is believed to be linked to the role of MYO6 in cellular migration [91,92].

## 5. HUM-4

The *hum-4* gene encodes a myosin with a very large (~300 kDa) heavy chain that is unique to *C.elegans*, and is the founding member of class XII [23]. The motor domain is not well conserved, with little homology to other myosin classes. The closest orthologue to HUM-4 in humans is a myosin of class XV, MYO15A and in Drosophila Myo10a. HUM-4 shares a 26% sequence identity to both human MYO15A and Drosophila Myo10a (Myo10a and MYO15a are 36% identical). Phylogeny analysis suggest a common origin for classes XII and XV [93]. Whilst the motor domain is less well conserved, the tail domain shows high degrees of similarity. Both myosins contain two myosin tail homology 4 (MyTH4) domains, a FERM (4.1 protein, Ezrin, Radixin, Moesin) domain, and a SH3 domain (Figure 2). Myosins of class XV are found in higher metazoan groups but not in nematodes, whereas class XII myosins are only present in nematodes, which is consistent with a common origin for class XII/XV myosins. Therefore, although belonging to a myosin of a different class, HUM-4 could be a valid model for studying the function, regulation and effect of mutations in the tail of MYO15A.

HUM-4 contains a unique 200 amino acid N-terminal extension, the function of which is unknown and with no shared homology to known domains. Another unique feature of this class of myosin is the position of the putative coiled-coil region at the carboxyl-terminal region of the tail. It has yet to be elucidated whether HUM-4 does form a dimer, but recent data suggests that human MYO15A is kinetically adapted for processive motility when oligomerised [94].

In humans, myosins of class XV were first discovered associated with congenital, recessive nonsyndromic deafness [95] and nearly 200 mutations in this gene were identified as causative of DFNB3 [96]. In humans, cochlear hair cells possess a bundle of actin-based stereocilia that detect sound. MYO15A is required for trafficking of essential compounds for stereocilia development, thus mutations in this myosin cause hereditary hearing loss. In *C. elegans*, HUM-4 is expressed in sensory neurons [33]. These neurons are essential for chemotaxis and mechanosensation, suggesting a potential role for this motor in regulating the worm’s response to its environment [97,98].

## 6. HUM-6

The 266 kDa HUM-6 protein is a myosin of class VII, and is an orthologue of human MYO7a, sharing a 53% sequence identity. In Drosophila a myosin of class VII called crinkled is expressed, which is 62% identical to human MYO7a [99]. The HUM-6 motor domain is followed by a neck region containing four IQ motifs (human MYO7a contains five IQ motifs), and a large tail domain containing two MyTH4 (myosin tail homology domain 4) and two FERM domains (Figure 2). Unlike human MYO7a, HUM-6 does not contain an SH3 domain.

In humans, MYO7a is widely expressed in the cochlea, retina, testis, lung and kidney [100,101,102], and interacts through the MyTH4-FERM domain in its tail to a number of different adaptor proteins and cell surface receptors such as cadherins and integrins thereby regulating cell–cell and cell-matrix adhesion [103]. In *C. elegans* the orthologue, HUM-6 is expressed in a number of different cell types, including the intestine which contains polarised cellular projections, suggesting an analogous function of HUM-6 to myo7a and its function in maintaining cellular projections [25]. HUM-6 is also expressed in the neurosecretory motor neuron and sensory neurons and the hypodermis [27,34].

Mutations in human MYO7a cause congenital deafness and blindness, clinically known as human Usher syndrome 1B [104] and non-syndromic deafness DFNB2 and DFNA11 [105,106,107,108]. Intriguingly, several of the causative mutations in human MYO7a are located at conserved residues also present in HUM-6, such as Asp218Asn in the motor domain [109] and Leu1087Pro in the tail [110], further highlighting the potential use of *C. elegans* to model human diseases.

## 7. HUM-7

The *hum-7* gene encodes a myosin of class IX [19]. Humans have 2 myosin genes of class IX—*myo9a* and *myo9b* while no MYO9 orthologue has been identified in *Drosophila*. HUM-7 is 34% identical to MYO9A and 30% to MYO9B. The domain organisation of myosins of class IX has several distinguishing features, including a Ras-association domain at the N-terminus of the protein (Figure 2). The motor domain has a number of unique mechanochemical properties, including a rate-limiting ATP-hydrolysis step and an unusually high-affinity for F-actin across different nucleotide states [111,112]. Also found in the head domain is a unique ~140 amino acid insertion, which interacts with actin filaments and contains an additional calmodulin-binding site. This insertion has been postulated to enable processive movement of the single headed MYO9 [113]. The neck region in HUM-7 and human MYO9B contains four IQ motifs, whilst human MYO9A has six IQ motifs. The tail region contains two atypical zinc ion-binding C1 domains in *C. elegans*, and one in both human MYO9A and MYO9B. A Rho GTPase activating protein domain is located at the C-terminus [114]. This domain catalyses GTP hydrolysis by small monomeric GTPases of the Rho subfamily, switching them from the active GTP-bound “on” state to the inactive GDP-bound “off” state.

Compared with the other unconventional myosins discussed above, a number of studies have been performed with HUM-7, providing insights into the unique properties of this motorised signalling molecule. Liao and colleagues demonstrated that HUM-7 moves processively towards the plus-end of actin filaments, which has not previously been observed with monomeric motors [113]. This processive movement could be due the unique insert, which acts as an actin tether [115]. It has also been shown that the rate-limiting step in the ATPase cycle of MYO9B is ATP hydrolysis, rather than phosphate release as in other myosins characterised to date [111]. These characteristics are likely due to the unique domain organisation of MYO9.

Defects in the activity of the two human MYO9 proteins are linked to a number of diseases. Mutations in MYO9A for example lead to Myasthenic Syndrome, a disorder characterised by altered transmission of signals from nerve cells to muscles [116]. It has been also been suggested that mutations in MYO9B are associated with a number of intestinal disorders, such as celiac disease, Crohn’s disease, ulcerative colitis and pancreatitis [117,118,119]. The mechanisms how loss of MYO9B function leads to pathogenesis in these disorders have yet to be determined. The genetic tractability of *C. elegans* makes it an ideal organism to study the impacts of mutations in MYO9 and its effects on Rho activity and associated cell signalling pathways.

In the nematode, HUM-7 has been shown to modulate RHO-1/RhoA activity during embryonic morphogenesis [35]. HUM-7 acts a GTPase-activating protein (GAP) for RHO-1/RhoA and CDC-42 GTPases. In this pathway HUM-7 is regulated by SAX-3/ROBO controlling F-actin dynamics through RHO-1/RhoA during epidermal cell migration in developing worms. Interestingly, this pathway is conserved in cultured human lung cancer cells [120], highlighting furthermore the potential of *C. elegans* as a model organism and further work may yield new insights into previously unknown diseases linked to altered HUM-7/MYO9A/B.

### C. elegans Myosins of Class II

The class II family of myosin motors were traditionally termed ‘conventional’ myosins. Class II myosins are hexameric complexes, composed of two heavy chains, two essential and two regulatory light chains. The light chains confer structural stability and regulation via phosphorylation, respectively. These molecular motors are characterised by their ability to assemble into thick filaments, low duty ratio (the time spent in the ATPase mechanochemical cycle strongly bound to actin), the ability of individual motor “heads” to operate independently of each other, and their rate-limiting phosphate release. Myosin II is not just found within muscle cells—non-muscle myosin II is ubiquitously expressed in various mammalian cell types. They have been implicated in a wide range of biological processes such as cell adhesion, cell migration, cell division and phagocytosis [44,121] to name just a few. The overall structure of the sarcomere, (the fundamental unit of contraction in which muscle myosin is found) is conserved from *C. elegans* to humans, and extensive work has utilised nematode to probe many processes involved in muscle development, muscle ageing and myopathies [122], the scope of which goes beyond this review. We will, however, provide a brief over view of isoforms of myosin class II in *C. elegans*.

Humans have 14 myosin class II genes, three of which are non-muscle myosin II and one which is a smooth-muscle specific isoform. *C. elegans* have four muscle myosin genes, termed *mhc-a*, *mhc-b*, *mhc-c* and *mhc-d*. They also have three non-muscle myosin genes (*nmy-1*, *nmy-2* and *nmy-3*), and two further genes *myo-5* and *myo-6* which have yet to be characterised [19]. *C. elegans* have both striated and non-striated muscles. Non-striated muscles include 20 pharyngeal muscle cells, two stomatointestinal muscles, one anal depressor muscle, one anal sphincter muscle, eight vulval muscles, eight uterine muscles, and 10 contractile gonadal sheath cells [122]. They also have 95 striated body wall muscle cells, which form a single layer of cells and are arranged in four longitudinal bands of two mutually offset rows of cells, named quadrants, running from head to tail. These muscles are the functional equivalents of vertebrate skeletal muscles. MHC-A and MHC-B proteins are the most abundant, and are found exclusively in the body wall muscle [36,37,123,124].

The high homology between *C. elegans* muscle components and their human counterparts has been utilised in numerous studies of myosin folding, particularly of the chaperone, UNC-45. UNC-45 (uncoordinated-45) was originally identified as a result of mutations causing structural disruption of thick filaments in body wall muscle in *C. elegans* [36,125,126]. The UNC-45 gene is essential in *C. elegans*, whilst missense mutations result in disorganized and reduced numbers of myosin-containing thick filaments giving rise to a slow-moving, or uncoordinated, phenotype of adult worms [127,128]. UNC-45 homologs are present throughout metazoans, including worms, flies, frogs, mice and humans [36,129,130,131,132]. Gazda and colleagues reported the crystallisation of the *C. elegans* UNC-45 protein [133], a key step in understanding chaperone function to fold myosin and assemble thick filaments. This work using *C. elegans* as model organism to garner insights into a long-standing question in myosin research, namely how myosin is incorporated into thick filaments, highlights some of the landmark discoveries the nematode can provide in the field.

Whilst *C. elegans* have proven a useful model for studying muscle folding, they have also been proposed to be suitable as a model system for a number of myopathies. MHC-B shares a 54% sequence homology to human beta-cardiac myosin, a protein which counts for ~30% of known missense mutations that give rise to dilated and hypertrophic cardiomyopathy [134]. The lack of a cardiac and circulatory system in *C. elegans* does limit a full-systemic model of cardiac disease. Despite this, it has been proposed that the nematode can be used to model protein–protein interactions in human cardiomyocytes because of the functional interactions in *C. elegans* body wall muscle [135]. The need to study the effect of these mutations on molecular function at the mechanochemical ATPase level cannot be overstated. Indeed, the nematode has been used to measure ATPase activity, force generation and motility in muscle contraction [136]. Mutations in *C. elegans* myosins have also been used to study myosin storage myopathy [137] and congenital myopathies [138], whilst drug screens have been successfully utilised in the nematode for RYR1-related myopathy [139].

## 8. Conclusions and Future Perspectives

Myosins are fundamentally important for directed movement on actin filaments, a process which is required for a wide range of cellular activities. The human genome has a repertoire of 12 myosin classes, all adapted to perform specific functions within the cell types they are expressed. Many of these myosins have orthologues to *C. elegans* myosin, as has been discussed in this review. Moreover, *C. elegans* is a well-established organism for the study of aging and longevity. The role of unconventional myosin motors in aging has yet to be explored. The conservation of endogenous locations and functions highlights the benefit of studying these unconventional myosin motors in such a well-established model organism.

The role of myosin motors in human disease makes it an attractive clinical drug target, fuelling the need to initiate further studies on this family of motor proteins. *C. elegans* is a well-established in vivo model system that has emerged as an extremely valuable resource in pharmacological drug discovery. To make potential use of *C. elegans* in a pharmacological screen for modulators of myosin activity, requires a more complete understanding of the cellular, biochemical, and biophysical characteristics of the *C. elegans* myosin homologues, which can form the basis of future work.

## Data Availability

Not applicable.
